# Sex differences in life expectancy in dementia, mild cognitive impairment, and subjective cognitive decline

**DOI:** 10.1093/geroni/igaf122.3190

**Published:** 2025-12-31

**Authors:** Rachel Amland, Bjorn Heine Strand

**Affiliations:** University of Oslo, Oslo, Oslo, Norway; Norwegian Institute of Public Health, Oslo, Oslo, Norway

## Abstract

**Introduction:**

It is unclear how dementia affects loss in life expectancy (LE). In this registry-based study, we aimed to study sex differences in LE and loss in LE in dementia, mild cognitive impairment (MCI), and subjective cognitive decline (SCD).

**Methods:**

16,358 patients diagnosed with dementia, MCI or SCD from the Norwegian registry of persons assessed for cognitive symptoms (NorCog) during 2009-2022 were included and followed up for mortality. Sex differences in LE, and loss in LE, were predicted using flexible parametric survival models and sex specific mortality in the general population as reference.

**Results:**

Women with dementia had the largest loss in LE; 17 years loss at 60 years, correspondingly men lost 13.5 years. Similar patterns were observed for MCI and dementia subtypes.

**Discussion:**

Women with dementia or MCI had larger loss in LE compared to men with these diagnoses.

